# Recent advances in metabolic engineering of microorganisms for advancing lignocellulose-derived biofuels

**DOI:** 10.1080/21655979.2022.2051856

**Published:** 2022-03-17

**Authors:** Abhishek Joshi, Krishan K. Verma, Vishnu D Rajput, Tatiana Minkina, Jaya Arora

**Affiliations:** a Laboratory of Biomolecular Technology, Department of Botany, Mohanlal Sukhadia University, Udaipur 313001, India; b Key Laboratory of Sugarcane Biotechnology and Genetic Improvement (Guangxi), Ministry of Agriculture and Rural Affairs/Guangxi Key Laboratory of Sugarcane Genetic improvement/Sugarcane Research Institute, Guangxi Academy of Agricultural Sciences, Nanning - 530007, China; c Academy of Biology and Biotechnology, Southern Federal University, 344090, Russia

**Keywords:** Biofuels, lignocellulose, microorganism, metabolic engineering, genome engineering

## Abstract

Combating climate change and ensuring energy supply to a rapidly growing global population has highlighted the need to replace petroleum fuels with clean, and sustainable renewable fuels. Biofuels offer a solution to safeguard energy security with reduced ecological footprint and process economics. Over the past years, lignocellulosic biomass has become the most preferred raw material for the production of biofuels, such as fuel, alcohol, biodiesel, and biohydrogen. However, the cost-effective conversion of lignocellulose into biofuels remains an unsolved challenge at the industrial scale. Recently, intensive efforts have been made in lignocellulose feedstock and microbial engineering to address this problem. By improving the biological pathways leading to the polysaccharide, lignin, and lipid biosynthesis, limited success has been achieved, and still needs to improve sustainable biofuel production. Impressive success is being achieved by the retouring metabolic pathways of different microbial hosts. Several robust phenotypes, mostly from bacteria and yeast domains, have been successfully constructed with improved substrate spectrum, product yield and sturdiness against hydrolysate toxins. Cyanobacteria is also being explored for metabolic advancement in recent years, however, it also remained underdeveloped to generate commercialized biofuels. The bacterium *Escherichia coli* and yeast *Saccharomyces cerevisiae* strains are also being engineered to have cell surfaces displaying hydrolytic enzymes, which holds much promise for near-term scale-up and biorefinery use. Looking forward, future advances to achieve economically feasible production of lignocellulosic-based biofuels with special focus on designing more efficient metabolic pathways coupled with screening, and engineering of novel enzymes.

## Graphical Abstract

**Figure:**
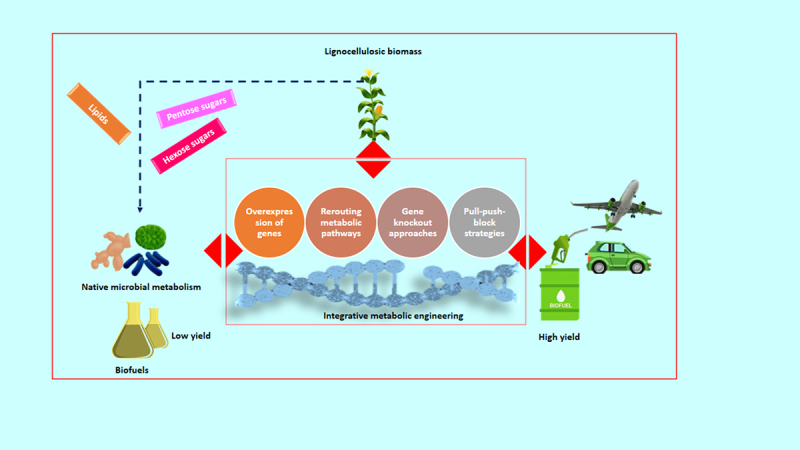

## 1. Introduction

Climate change, environmental degradation, and pollution-related health problems in the population have all been linked to the increasingly excessive consumption of fossil fuels by the transportation sector, globally [1]. These issues have diverted industry, decision-makers, and scientists’ attention towards generating alternative fuels from renewable sources [2,3]. The United Nations (UN) has also introduced affordable and clean energy as a prime concern among Sustainable Development Goals (SDG) and set different targets to make it available for everyone without competing for future generations [4]. Most recently, an inclusive focus was given on greener transport vehicles to cut down the net carbon emissions and global warming in the 26^th^ annual summit of COPs (Conference of the Parties) held in Glasgow, United Kingdom [5].

Biofuels primarily produced using bio-based materials, i.e., starch, sugar, lignocellulose, animal fats, and other biopolymers, are considered cost-effective and environmentally benign alternatives to petroleum-based fuels impoverishment [6,7]. Lignocellulose is deemed the cheapest and most abundant biopolymer among the available bio-based materials on the earth [8,9]. Several studies have reported bioethanol, biodiesel, and other valuable petrochemicals, that is, butanol, isopentanol, terpenes, etc., production from lignocellulosic biomass (LCB). Its feasibility has been hampered by the inherent recalcitrance of the biomass [10–12]. The increased understanding of cell wall polysaccharides and/or lignin biosynthesis and recent advances in metabolic engineering have enabled the possibility of increased digestibility of the LCB with the improved release of fermentable sugars. Lipid biosynthesis and storage engineering are also advantageous to enhance biofuel production [13,14].

**Figure 1.**
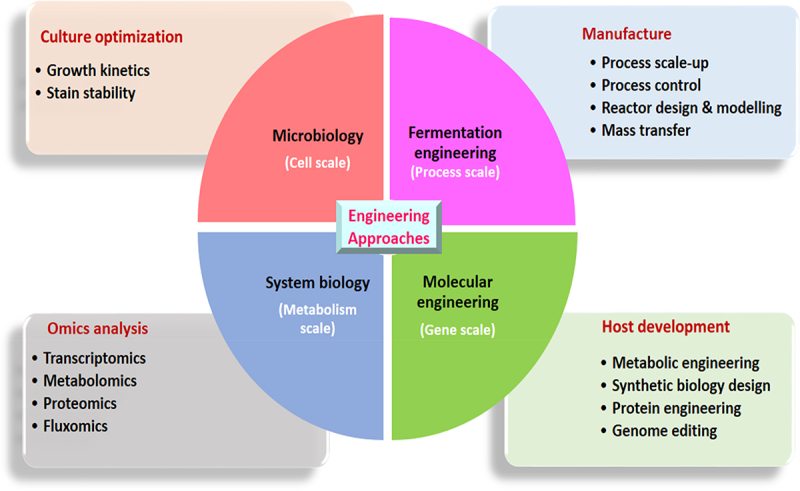
Different levels of engineering approaches at microbes for enhanced production of Biofuels.

The microbial-assisted bioconversion processes greatly affect the cost-competitive biofuel production [15]. It seems that the capital and operational cost associated with the microbial bioconversion process are the highest contributors to the overall cost production of biofuels [16]. Several native and non-indigenous microorganisms can catalyze lignocellulose substrates (LCS) into a broad array of biofuels. Still, its feasibility directly relies on the inherent metabolic facet of that particular microorganism [17,18]. Nevertheless, some of these substances or their precursors can also be synthesized from distinct metabolic pathways occurring naturally in microorganisms. The ideal microorganism/strain for sustainable scale-up and biorefinery use should utilize various substrates and produce a higher yield of end products and better sturdiness against inhibitors [19]. These metabolic shortcomings can be addressed by tailoring or redesigning the metabolic facets of each microbe/stain. Substantial progress has been made in this regard during the last couple of years. Numerous engineering strategies were employed to design and optimize pathways in microorganisms for advancing biofuel production have been reconnoitred in Figure 1. Furthermore, these approaches would help to improve the substrate spectrum and metabolic fluxes towards biofuel pathways, as well as enhance the possibility of high-yielding phenotypes [20–22]. In addition, cell surface display engineering has enabled precise modification of surface enzymes to enhance the biocatalytic capabilities of microbial cells. This contributes greatly to the expansion of biofuels production from lignocellulose substrate [23,24]. Through genome editing, the ability to create new robust industrial phenotypes with high volumetric productivity, improved substrate utilization, and increased inhibitor tolerance has been demonstrated [25,26]. Recent efforts to develop genetically modified microorganisms or recombinant strains are under intense development. A number of microbial platforms and strains with actual scale-up features have been patented or are in the process of being accepted [27–29].

This paper aims to review the metabolic engineering strategies used to produce robust microbial workhorses that use LCB as the primary feedstock for biofuel production. In recent years, there have been a number of breakthroughs in this holistic approach, mainly focusing on the different microbial domains and their metabolic pathways that produce biofuels. These are described in detail in this article, along with considerations of future development opportunities.

## 2. Recent metabolic advances in the lignocellulosic feedstock

### 2.1. Cell wall engineering

The natural recalcitrance of LCB is a considerable barrier to biofuel sustainability. To overcome this problem, researchers have previously focused on disrupting structural and compositional complexity through distinct strategies, such as pre-treatments and consolidating bioprocessing [30,31]. However, such methods effectively solubilize the lignocellulose polymers into fermentable sugars but employed several intricacies, that is, destruction of valuable sugars fraction, generation of inhibitor compounds, and irreversible salts, which can make downstream processes complex and expensive [16,32]. Recent advances in metabolic engineering have enabled the altered synthesis of the various wall polymers to increase polysaccharide accessibility and reduction of polymer-derived processing inhibitor, along with high hexose/pentose ratio in the biomass [33].

Cellulose is a major homopolysaccharides in the plant cell wall. Additionally, it consists entirely of glucose, an indispensable substrate for producing biofuels [34]. Significant efforts have been devoted to cellulose biosynthesis and remodeling in the lignocellulose feedstock for the purpose of cost-effective biofuel production. The expression regulation of genes including cellulose synthase (CesA), sucrose synthase (SuSy), glycosyltransferases (GT), and glycosyl hydrolases (GH) has resulted in drastic changes in cellulose content, crystallinity, and total biomass as well [35–37]. Recent work in transgenic poplar has demonstrated that overexpression of PdDUF266A dramatically improved cellulose content (up to 37%) and total biomass (17–34%) than wild type [38]. Furthermore, the cellulose depolymerization and ratio of the total sugar release after enzymatic saccharification were also increased up to 13% and 38%, respectively, as compared to control. In a different investigation, overexpression of the PsnSuSy2 gene (derived from hybrid poplar) in tobacco increased its cellulose content by 18%, and decreased cellulose crystallinity by 11%, when compared with control [39].

Similarly, the genes involved in synthesizing mixed-linked glucan have been explored to improve hexose contents as β-1, 3 bonds can prevent the aggregation of cellulosic components and render the cell wall more digestible. Recent work has shown that overexpression of cellulose synthase-like F6 (from rice) gene in *Arabidopsis* can increase glucose accumulation in the matrix cell wall fraction by four times and saccharification by up to 42% compared to control lines [40]. In a different investigation, overexpression of UDP-rhamnose/UDP-galactose Transporter1 (URGT1) together with galactan synthase (GalS1) and glucose epimerase (UGE2) increased stem galactose levels to four times than wild-type plants [41].

Also, some recent efforts targeting xylan backbone (comprises a linear chain of b-1, 4-D-xylosyl residues) to alter recalcitrance and increase saccharification efficiency, but there are trades in many cases offs to be considered [13,42]. *In planta*, the expression of various enzymes involved in xylan biosynthesis (i.e., acyltransferase, ferulic acid esterases, etc.) can be the attractive targets to facilitate the biomass saccharification efficiency [43,44]. Recently, overexpression of the rice OsAT10 (a BAHD acyltransferase gene) in switchgrass enhances saccharification efficiency up to 40% [45]. Similarly, OsAT10 overexpression increases saccharification efficiency in sorghum OsAT10 lines [46].

Lignin, the end product of the phenylpropanoid pathway, is the foremost cause behind the inherent recalcitrance of the LCB [47]. Recently, substantial progress has been made to manipulate the genes encoding enzymes of the phenylpropanoid pathway, such as phenylalanine ammonia-lyase, cinnamate 4-hydroxylase, hydroxycinnamoyl-CoA shikimate/quinate, coniferyl aldehyde dehydrogenase, cinnamyl alcohol dehydrogenase, laccase, and more [48]. The overexpression or down-regulation of related genes significantly reduces the lignin contents and facilitates the lignocellulose saccharification efficiency [49–51]. Recently, overexpression of rPvGRF9 genes significantly increased per plant sugar yield with less lignin content in switchgrass [52].

Nevertheless, these recent efforts help to modulate plant cell wall components considerably. Still, its compositional disparities among the species and tissue often need to be optimized or redesigned to facilitate lignocellulose quality for conventional and future biorefinery use.

### 2.2. Lipid engineering

The increased understanding of lipid biosynthesis and storage has also enabled the possibility of cost-effective biofuel production, primarily biodiesel, and more recently jet fuel [53]. Lipids are energy-dense biomolecules frequently stored as triacylglycerols (TAGs) in the plants’ seeds and non-seed tissues (vegetative tissues). The TAGs currently utilized for biodiesel production are mainly derived from edible seed feedstock. Several food crops, such as corn, soybean, oilseed crops, that is, *Camelina sativa, Jatropha curcas*, etc., have been established for high seed oil content thorough engineering of lipid biosynthetic pathways and genes [54,55]. At the same time, established pre-eminence of food versus fuel debates, it is unlikely the TAGs production from current oilseed feedstock will contribute substantially to the biofuels industry in the future. The vegetative tissues (i.e., leaf, stem, etc.) that constitute most of the plant biomass are also capable of synthesizing TAGs, but their amount typically accounts for only a minor portion of total lipids [56,57].

For the last couple of years, metabolic engineering to increase the TAGs biosynthetic capacity among vegetative tissues has attracted wider attention as a sustainable oil/lipid yielding platform for lignocellulosic biorefinery [58]. The attempts to encourage the production of TAG in vegetative tissue have mainly focused on the integrated concept (pull, push, package, and protect) of single or multiple genetic interventions [59–69], resulting in augmented TAG levels with better compositional facets in distinct non-food crops (Table 1). However, in a model plant, *Tobacco*, the highest TAG yield (30–33% DW) to date was achieved by combinatorial optimization of TAG biosynthetic pathways [67], but it is not always straightforward as these pathways are highly conserved across different species [70].

**Table 1.** Summary of some important metabolic engineering approaches to increase oil/lipid synthesis in vegetative tissues in non-food crops

Targeted trait	Metabolic engineering strategies	Source organism	Target plant	Outcomes	References
Oil/TAG accumulation, assembly and other stacking approaches	Ectopic overexpression of diacylglycerol acyltransferase 2(*DGAT2*)	*Cyperus esculentus*	Tobacco	7.15- fold increase in TAG with 31.33%oleic acid content	[59]
	Transient overexpression of acyl-lipid thioesterases (ALT) 1–4	*Arabidopsis thaliana*	Tobacco	Increased accumulation of 12–14 carbon-length fatty acids and 6–8 carbon-length fatty acids in leaves	[60]
	Combined co-expression of wrinkled 1*(WRI)* and *DGAT1* with Oleosin L(*OLEl*)	*Arabidopsis thaliana* and *Sesamum indicum*	Tobacco	Up to 2.3-fold increase in oil	[61]
	Constitutive co-expression of *WRI1, DGAT1-2*, cysteine-oleosin; and ribonucleic acid interference (*RNAi*)-suppression of sugar-dependent1(*SDP1*)	*Sorghum bicolor*	Sugarcane	4.3% (DW) TAG in stem and 13.0% (DW) in leaves	[62]
	Transiently expressing of cyclopropane fatty acid synthases (*CPFASes*) and *DGAT*	*Bacteria*	Tobacco	15% increased dihydrosterculic acid content in TAG	[63]
	Combined overexpression WR*1*1, *DGAT2a* andoleosin-L(*OLEl*)	*Zea mays, Umbelopsis ramanniana*and *Sesamum indicum*, respectively	*Sorghum bicolor*	3% to 8.4%(DW) TAG in leaves with reduced transitory starch and soluble sugar levels	[65
	Ectopic overexpression of *DGAT1*a	*Vernonia galamensis*	Tobacco	3.5–5.0-fold increase up to 9% (DW) with enhanced linoleic acid and reduced α-linolenic acid	[65]
	Transient expression of*WRI*1	*Ricinus connunis*	Tobacco	4.3–4.9 fold increased oil content	[66]
	Silencing of *SDP1* lipase and overexpression of leafy cotyledon 2 *(LEC2*) transcription factor	*A.thaliana*	Tobacco	30–33%(DW) TAG in leaves	[67]
	Transient expression of *WRI1A,B*, or *C*	*Camelina sativa*	Tobacco	2.5- to 4.0-fold increased TAG in leaves	[68]
	Constitutive co-expression of *WRI*1, *DGAT*1-2 and *OLE*1 and simultaneous co-suppression of ADP-glucose pyrophosphorylase (*AGP*ase) and a subunit of the peroxisomal ABC transporter1 (*PXA*1)	*A. thaliana*	Sugarcane	Increased TAG accumulation in leaves or stems by 95- or 43-fold to 1.9% or 0.9% of dry weight (DW), respectively	[69]

Several avenues might be explored for utilizing vegetative tissues as high-yielding TAG platform for biofuels, including (a) modulation of lipid transporters (i.e., plastidial lipid transporter TGD5 (trigalactosyldiacylglycerol5) and fatty acid exporter 1 (FAX1); peroxisomal transport protein CTS (comatose), etc.) to optimize the lipid fluxes between the subcellular compartments, (b) development of more specific promoters (i.e., vegetative tissue-specific, developmental stage-specific, etc.) to maximizing the unusual fatty acids yields and minimizing the adverse phenotypic effects, (c) engineering of lipids metabolism and lipid droplets (LDs) for accumulation of high-value neutral lipids, that is, wax esters, volatile lipids, etc., and to enhance the energy density over LDs through the engineering of extracellular surface (cuticle) of a given plant without any adverse effects on plant growth performance [71–73].

However, a recent techno-economic analysis proposed that engineered hemp (*Cannabis sativa*) with a minimum of 10% lipid content can produce up to 326 gallons of total biofuels per hectare of agricultural land than soybean with the production cost of USD 4.13/gallon [74].

## 3. Overview on major metabolic pathways of microorganism for biofuel productions

Both pentose and hexose sugars (i.e., xylose, arabinose, glucose, etc.) derived from the LCS are the prime carbon source for the production of biofuels by industrially tractable microbes, such as yeasts, *Escherichia coli, Clostridium* spp., and more [75]. These microorganisms have distinct catalytic enzymes and display specific metabolic paths to produce distinct biofuels, including higher alcohols and hydrocarbons [76,77]. In general, microbes use xylose and glucose as initiating molecules for the anaerobic synthesis of ethanol via Entner-Doudoroff (ED-P) and Embden-Mayer Hoff-Parnas (EMP-P) pathways [78]. The higher alcohols include linear-chain alcohols and branched-chain alcohols can be synthesized via either fatty acid or amino acid pathways. Besides, fatty acid metabolism is imperative for producing linear-chain alcohols, while amino acid metabolism is advantageous for branched-chain alcohols [79].

**Figure 2.**
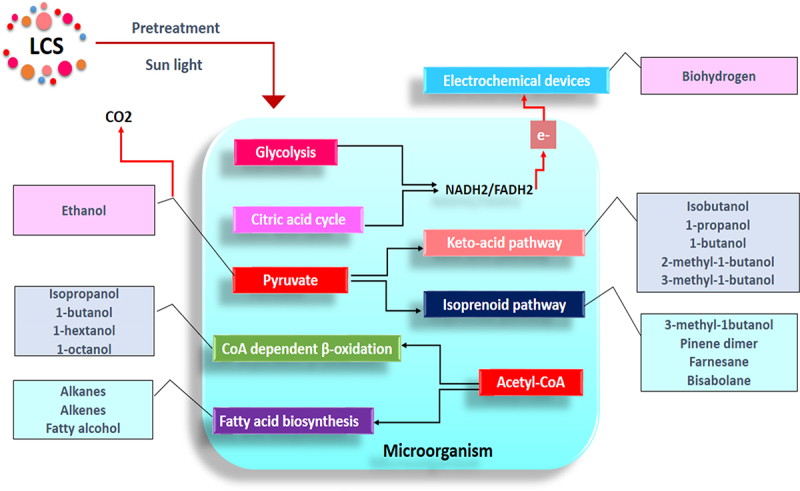
Overview of major metabolic pathways of microorganisms for biofuel productions.

Several detailed reports have been published on molecular engineering and system biology strategies employed to design and optimize biofuel production microbial metabolic pathways [80–82]. Much of the reports highlighted that the advanced biofuel production could be achieved mainly through four major metabolic pathways: (1) the keto-acid pathway, (2) the isoprenoid pathway, (3) fatty acid synthesis pathway, and (4) CoA dependent β- oxidation pathway or reverse b-oxidation pathway. Pyruvate is the precursor molecule for initiating the keto-acid pathway used to produce isobutanol, 1-propanol, 2-methyl-1-butanol, and more. It is also a precursor molecule of the isoprenoid pathway used to produce 1-isopropyl-4 methylcyclohexane, pinene dimer, farnesane, and bisabolane. Similarly, acetyl-CoA is the precursor molecule for initiating the fatty acid synthesis pathway and CoA-dependent β- oxidation pathway. The fatty acid synthesis pathway is advantageous for producing alkanes, alkenes, and fatty alcohols, while the CoA-dependent β- oxidation pathway is for isopropanol, 1-butanol, and more (Figure 2). Most recently, the polyketide biosynthetic pathway mediated by polyketide synthases (PKSs) and cognate thioesterase (TE) has also been tweaked for the production of alkanes and alkenes [60,83].

## 4. Recent metabolic advances in microorganism for biofuel production

### 4.1. Bacteria

Bacteria are promising hosts for the generation of a variety of fuel-like compounds. These have also been given preference to metabolic pathways manipulation because of their substrate suitability, fast growth rate, and well-tractable physiology and genetics. The user-friendly hosts, that is, *Zymomonas mobilis* and *E. coli, Clostridium*, etc., have been the center of recent metabolic engineering studies to develop a model microbial chassis for sustainable biorefinery practices in the modern era [84].

The *Z. mobilis* is a natural ethanologenic bacterium with excellent industrial features like high carbon substrate uptake rates, high ethanol tolerance (up to 16% v/v), and a low aeration cost due to anaerobic exposure fermentation [85]. The main drawback of native *Z. mobilis* is its inability to utilize pentose sugars for ethanol production. Several recent metabolic engineering efforts have been undertaken to broaden the substrate spectrum of *Z. mobilis* via the introduction and efficient expression of heterologous genes coupled with utilization and assimilation of pentoses, such as xylose and arabinose. Some efficient pentose utilizing strains have been developed through combining metabolic engineering and adaptive laboratory methods. These recombinant strains reportedly harbored improved xylose utilization, co-utilization of mixed sugars and enhanced ethanol production [86–88]. In addition, xylose transport has also been identified as a major barrier to the efficient utilization of xylose by *Z. mobilis*. The native *Z. mobilis* has been reported to transport xylose via Glf (Glucose facilitated diffusion protein) transporter. The Glf has a high affinity for glucose, causing competitive inhibition of xylose uptake in mixed sugar supplemented medium [89]. Recently, some xylose-specific transporters, that is, XylE, and ABC type transporter, were successfully introduced in *Z. mobilis* to enhance the rate and extent of xylose uptake [90,91]. An experiment demonstrated , recently, that overexpression of ABC type transporter enhances the rate of xylose utilization by 48.9% as compared to parental stains in the presence of glucose [91]. Recent efforts in *Z. mobilis* engineering have also enabled several strains to tolerate furfural and acetic acids, which are the predominant toxic inhibitors during lignocellulosic ethanol production [92,93]. Nouri et al [94] developed recombinant strains with improved tolerance against the multiple inhibitors by overexpressing the genes hfq (a transcription regulator) and sigE (a transcription factor). Furthermore, strain engineered to express sigE also showed 2-fold and 4-fold higher ethanol yields than the hfq-overexpressing strain and parental strain, respectively. Recently, a dioxygenase coding gene (ZMO1721) was overexpressed to develop a recombinant strain resistant to many phenolic aldehydes, including 4-hydroxybenzaldehyde, syringaldehyde, and vanillin[95].

Other than ethanol, *Z. mobilis* possesses endogenous abilities to produce other fuel alcohols, such as isobutanol and 2, 3-butanediol. For the 2, 3-butanediol production, recombinant strains were successfully developed by expression of acetolactate synthase, acetolactate decarboxylase, and butanediol dehydrogenase encoding genes [96,97]. The best-enhanced strain was able to produce more than 10 g/l of 2, 3-butanediol from glucose and xylose as well as sugar streams derived from deacetylation and mechanical refining [96]. Some recombinant strains have recently been developed for isobutanol production by the regulation of alcohol dehydrogenase (adhA) and 2-ketoisovalerate decarboxylase (kivd2) [98]. Recently, a maximum of 4.0 g/l of isobutanol was achieved by the overexpression of gene kivd2 combined with a synthetic heterologous operon als-ilvC-ilvD[99].

*E. coli* is another bacterium that has been extensively investigated for biofuel production. It is naturally capable of utilizing both pentose and hexose, and is amenable to produce ethanol by an endogenous ethanologenic pathway. A disadvantage of this pathway is that only 1 mole of ethanol can be produced from 1 mole of glucose [100]. This yield is relatively inefficient as compared to the homeoethalogenic pathways of *Zymomonas* and *Saccharomyces* species. Due to this, initial engineering effort has been devoted to modify existing *E. coli* pathways for ethanol production [101]. Several recombinant strains have recently been developed by introducing relevant foreign genes, such as pyruvate decarboxylase (pdc), alcohol dehydrogenase (adh), and more. The resulting engineered strains were able to effectively direct carbon flux to higher ethanol production [102]. The glycolytic pathway of *E. coli* KO11 was recently modified through knocking out phosphoglucose isomerase (pgi) to redirect carbon flux from glucose via ED-P and PP-P pathways [103]. (Huerta‐Beristain et al. 2017). Further engineering KO11 by the adaptive evolution and deletion of pta, ack, and ldh genes provided the strain KO11 PPAL, which led to higher yield of ethanol. Recently, the intracellular NADH/NAD^+^ ratio in the *E. coli* KO11 was improved by the overexpression of formate dehydrogenase from *Mycobacterium vaccae*. The enhanced stains produced more than 36% ethanol as the original KO11 strain in culture for 24 h [104]. Apart from this, simultaneous utilization of both xylose and glucose is also a considerable barrier towards effective ethanol production. In recent research, *E. coli* MG1655 was engineered through the combined genetic and evolutionary engineering to utilizing cellobiose and xylose simultaneously. The recombinant strain was capable of utilizing approximately 6 g/L of cellobiose and 2 g/L of xylose in approximately 36 h [105]. A strain GX 50 was also engineered by transposon-mediated mutagenesis and metabolic evolution to enhanced xylose utilization in the presence of glucose [106].

Recently, recombinant E. *coli* B0013-2021HPA was engineered through introducing a mutation to pyruvate–glucose phosphotransferase system (ptsG). The enhanced strain was able to utilize almost all xylose, galactose as well as arabinose from lignocellulose hydrolysates and efficiently convert complex substrate mixtures to ethanol at 42°C under oxygen-limited fermentation conditions [107]. Recent effort has also been devoted to engineered *E. coli* strains with marked tolerance against the hydrolysate toxins, ethanol, and byproducts [108–110]. In recent research, *E. coli* strain LY180 was constructed by introducing multidrug resistance pumps, such as SugE and MdtJI [111]. The recombinant stain reportedly harbored furfural efflux and improved ethanol production in the presence of furfural or 5-hydroxymethylfurfural. More recently, a recombinant stain was developed through the combined adaptive laboratory evolution and CRISPR-enabled trackable genome engineering. The evolved strain could tolerate up to 4.7 g/L furfural and also exhibited marked cross tolerance towards end products including isobutanol, butanol, and ethanol as well as NaCl, and high temperatures [112].

In addition to ethanol, recent metabolic engineering efforts have also been devoted to develop *E. coli* strains for the production of isobutanol, isopropanol, and other bioalcohols. Several recombinant strains have been recently constructed with a goal of enhanced bioalchohals yield and tolerance [113,114]. Recently, a redesigned quorum sensing system combined with a metabolic toggle switch (QT-MTS) has been employed within *E. coli* model to redirect metabolic flux toward the target synthetic pathway [115]. The strain harboring MTS was reported to have 26-folds improvement in the intermediate pyruvate and final isobutanol production titer as compared control strain.

*Clostridium sp* have also been metabolically engineered for the production of butanol and higher alcohols [116]. Recently, wild *C. acetobutylicum* strain was engineered by the disrupting hydrogenase gene hydA. The recombinant strain was able to produce 18.3% more butanol with by-product acetone decreased by 31.2%. Further exogenous supplementation of methyl viologen enhanced the butanol yield up to of 0.28 g/g from the corn stover [117]. In a different investigation, recombinant *Clostridium cellulovorans* adhE2 strain was engineered through the introduction of thiolase (thlACA) from *C. acetobutylicum* and 3-hydroxybutyryl-CoA dehydrogenase (hbdCT) from *C. tyrobutyricum* [118]. The engineered *C. cellulovorans* strain was able to n-butanol from cellulose at a 50% higher yield (0.34 g/g). In addition, *Clostridium* spp. have also been metabolically engineered to produce biohydrogen [119]. Recently, recombinant *C. acetobutylicum* strains were developed by the overexpression of glucose-6-phosphate dehydrogenase and FeFe hydrogenase. The engineered strain was able to produce more than 1.4-fold higher ethanol yield than the wide type [120].

The fastest-growing bacterium, *Vibrio natriegens*, is recently engineered for heterologous production of 1, 3-Propanediol from glycerol. Systematic engineering of cellular transcriptional regulators and glycerol oxidation pathway leads to the 2.36 g/L/h output production of 1, 3-Propanediol [121]. The *Cupriavidus necator* H16 is an attractive living system, which can be metabolically engineered coupled with a gene knockdown process for directing carbon flux away from producing Poly[(R) −3 Hydroxybutyrate] and resulting in the production of biofuels like products [122]. Similarly, in *Clostridium ljungdahii*, an artificial isopropanol producing pathway was constructed through which this bacterium can efficiently use syngas and coproduce isopropanol 3-hydroxybutyrate and ethanol [123].

Methanotrophic bacteria are also an important source of bioproducts for biofuel production. Systems metabolic engineering of such bacterium has led to the advancement in methane-based bio-manufacturing of biofuels [124,125]. Some recent achievements by bacterial engineering are summarized in Table 2.

**Table 2.** Summary of major achievements in increasing biofuel production by engineering bacterial pathways

Bacteria species/ strain	Metabolic engineering/pathway	Targeted metabolite/substrate	Product	References
Caldicellulosiruptor bescii	Glycolytic pathway	Lignocellulose	Hydrogen	[236]
Clostridium acetobutylicum	Clostridial acetoacetyl-CoA-derived pathway	Glucose, starch and stover	n-Butanol	[237]
Clostridium autoethanogenum	Ferredoxin oxidoreductase pathway	Synthetic medium	Ethanol	[238]
Clostridium cellulolyticum	CoA-dependent pathway	Cellulose	n-butanol	[239]
Clostridium tyrobutyricum	Xylose metabolic pathway	Glucose and xylose	n-Butanol	[240]
Clostridium thermocellum	Embden-Meyerhof pathway	Cellulose	Ethanol	[241]
Corynebacterium glutamicum	Glycerol biosynthetic pathway	Glucose, xylose	3-Hydroxypropic acid	[242]
Enterobacter cloacae	Pentose phosphate pathway	Lignocellulose	2,3-Butanediol	[243]
Escherichia coli	Alginate metabolism pathway	Alginate, mannitol	Ethanol	[244]
	MEV pathway	Farnesyl pyrophosphate	Farnesol/ farnesene	[168]
	Ethanol biosynthetic pathway	Pyruvic acid	Fatty acid ethyl esters (FAEE)	[245]
Klebsiella pneumonia	Meso-2,3-butanediol synthesis pathway	Glucose	2-Butanol	[246]

### 4.2. Cyanobacteria

Cyanobacteria are the photosynthetic prokaryotes with more potent photosynthetic metabolomics than land plants. This metabolomics is advantageous to make them potential cell factories, which can efficiently produce biofuels and bio-based chemicals through sequestering of carbon. Also, its well-tractable physiology and more specific growth requirements attract attention to be engineered for industrial purposes [126].

Cyanobacteria are a good choice for solar-powered bioethanol production due to the presence of ethanologenic pathway [127]. Consequently, sufficient efforts have been devoted to engineering the existing pathways of some model cyanobacteria species, that is, *Synechocystis* and *Synechococcus* for improved ethanol production [128,129]. Several recombinant strains have been developed by employing the gene dosage, induced expression as well as cassette optimization. These engineered strains were able to produce up to 0.5 g/l ethanol per day [130–132].

In recent research, recombinant *Synechocystis* PCC 6803 strains were constructed through induced expression of four Calvin-Benson-Bassham (CBB) cycle enzymes, i.e., transketolase (TK), ribulose-1, 5-bisphosphate carboxylase/oxygenase (RuBisCO), aldolase (FBA), and fructose-1, 6/sedoheptulose-1, 7-bisphosphatase (FBP/SBPase) coupled with ethanol synthesis enzymes PDC and ADH [23]. The engineered strain that overexpressed the CBB cycle enzymes was able to produce 37–69% more ethanol and 7–10% more total biomass than the strain that expressed only the ethanol biosynthesis pathway. In a different investigation, ethanol production of the *Synechocystis* PCC 6803 was increased by 2-9-folds by combined expression of multiple native CBB enzymes [133]. More recently, a recombinant *S. elongatus* PCC7942 strain was developed through induced expression the ictB, ecaA, and groESL, and pdc-adhII genes [134]. The engineered strain was able to improve ethanol production and cell growth under a stimulated flue gas consisted CO_2_ (25%), SO_2_ (80–90 ppm) and NO (90–100 ppm).

Recent efforts have also been directed towards engineering cyanobacteria for the production of isobutanol and 1-butanol. Numerous recombinant strains of *Synechocystis* and *Synechococcus* were recently constructed through the introduction, overexpression, and evaluation of different genes related to isobutanol and 1-butanol biosynthesis [135,136]. Recently, a recombinant *Synechocystis* PCC 6803 strain was developed through the introduction of the genes kdc (keto-acid decarboxylase) and adh under the control of the CcaS/CcaR system. The enhanced strain was able to produce 238 mg/l of isobutanol and 75 mg/l of 3-methyl-1-butanol under red and green light illumination in 5 days [137]. In a different investigation, the introduction of NADH-dependent nitrate assimilation in *Synechococcus* PCC 7002 significantly enhanced the photosynthetic production of 2-methyl-1-butanol and isobutanol [138]. Also, metabolic profiles of high salinity stress engineered *S. elongatus* revealed that enhanced isobutanol production was caused by lipid degradation with the increase in NADH. The engineered strain proved to be a practical and feasible system for cost-effective isobutanol production [139]. Another strain of *S. elongatus* PCC 11801 has recently evolved in laboratory conditions through adaptive tolerance to higher concentrations of butanol [140]. Notwithstanding the above-mentioned advances, cyanobacterial bioethanol and higher alcohols require further improvement to become economically viable at an industrial scale.

The *Synechocystis* has recently been metabolically engineered to produce 1-Octanol, one of the emerging precursor molecules for high-value product formation, including fuel. The most efficient computational tools have been proved to be useful in determining appropriate cleavage sites of thioesters in the above cyanobacteria, which further play an important role in selecting 1-octanol [141]. Similarly, isobutene (a gaseous fuel) production in *Synechocystis* sp. PCC 6803 has been enhanced by the introduction of the α-ketoisocaproate dioxygenase gene from *Rattus norvegicus* (RnKICD), which resulted in optimization of the isobutene pathway [142]. Also, the industrial scale-up of terpenoids through sequential heterologous expression of bottleneck enzymes of Methylerythritol 4-phosphate pathway in *Synechocystis* sp. PCC 6803 has been achieved by Rodrigues and Lindberg [143].

In addition, large number of cyanobacteria are naturally capable of producing biohydrogen, which can be mainly synthesized by: (a) the action of bidirectional or reversible hydrogenase and (b) as a by-product of nitrogen fixation via nitrogenases [144]. Many novel hydrogen-producing strains have been obtained under various culture conditions in recent years [145,146]. Numerous efforts have been attempted to improve H_2_ yield by modulating both nitrogenase and hydrogenase-based H_2_ production systems [147,148], but it requires further improvement for economic H_2_ production at the industrial scale. Table 3 summarizes other efforts performed to improve the biofuel production potential of different cyanobacteria.

**Table 3.** Major engineering efforts performed to improve the biofuel production capacity of different cyanobacteria species/strains

Cyanobacteria species/ strain	Metabolic engineering/pathway	Targeted metabolite/substrate	Product	References
Anabaena sp.	AAR/ADO alkane biosynthesis pathway	Acyl-acyl protein reductase (AAR) and aldehyde decarbonylase (ADO)	Heptadecane	[247]
Methylobacterium extorqens	Ethyl malonyl-CoA pathway	Ethylamine	1-Butanol	[248]
Nostoc punctriforme	Hydrothermal liquefaction	Sugars, glucose	Liquid hydrocarbons (bio-oil)	[22]
Synechocystis sp.	Mevalonate pathway	Dimethylallyl pyrophosphate (DMAPP) and isopentenyl pyrophosphate (IPP)	(E)-α-bisabolene	[143]
	Ehrlich pathway	Glucose	Isobutanol	[249]

### 4.3. Yeasts

Yeasts, including native, thermotolerant, halophilic, etc., have attracted much attention as cell factories convert lignocellulose to biofuels and bio-products. The large spheroidal cells, simple bioprocessing, minimal nutritional requirements, and better inhibitor tolerance relative to many bacteria and cyanobacteria can expand this scenario [149]. Although *Saccharomyces cerevisiae* has been the promising host for ethanol production from the earliest time, it cannot efficiently utilize the sugar polymers, such as xylose, and cellobiose available in LCB [81]. Also, glucose repression of xylose fermentation and toxic fermentation inhibitors in lignocellulosic hydrolysates adversely affects productivity and yield. Despite the competitive production costs and ideal utilization of substrate, rapid sugar metabolism after eliminating ethanol production is advantageous to producing advanced biofuels and biochemicals [150].

**Figure 3.**
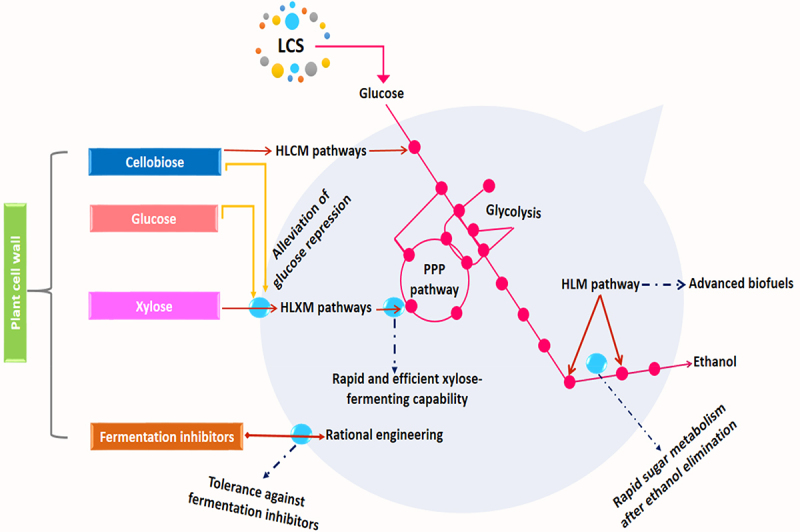
Metabolic engineering of yeast (*S. cerevisiae*) for the production of biofuels from lignocellulosic sugars, PPP = pentose phosphate pathway, HLXM = heterologous xylose metabolic pathways, HLM = heterologous metabolic pathway, HLCM = heterologous cellobiose metabolic pathways.

Several metabolic engineering strategies to address the issues mentioned above have been undertaken and implemented to yeast in the last couple of years (Figure 3). For instance, engineering the PPP and XAP pathways (either oxidoreductase or isomerase-based) has been reported to maximize xylose utilization and alleviate glucose repression on xylose fermentation [151]. Hoang et al. [152] constructed an efficient xylose-fermenting *S. cerevisiae* strain through combinatorial CRISPR–Cas9-mediated rational and evolutionary engineering. This isomerase-based xylose-fermenting strain, named XUSE, demonstrates the efficient conversion of xylose into ethanol with a high yield of 0.43 g/g and exhibited simultaneous fermentation of glucose and xylose with negligible glucose inhibition. Significantly, higher ethanol yields were also achieved by engineering the high osmolarity glycerol pathway [153]. Recently, the highest ethanol yield (0.492 g/g total sugars) from lignocellulosic hydrolysates was achieved by overexpressing a mutant SFA1in *S. Cerevisiae* [154].

Studies also focused on transporter and transcription factor engineering to develop more powerful ethanol-producing strains as yeast lacks xylose-specific transporters. Some novel xylose transporter, such as XltA (from *Aspergillus niger*) and Xut3 (from *Scheffersomyces stipitis*), were successfully introduced into yeast to maximize xylose uptake [155]. Soon after, the directed evolution of xylose-specific transporter AN25 from *Neurospora crassa* was found suitable for improved xylose transportation and glucose-xylose co-utilization [156]. Improved xylose uptake and consumption with higher ethanol yield were obtained through the directed or adaptive evolution of Nuclear-Localized Hexokinase 2(HXK2) and endogenous hexose transporters (HXT2) and CYC8 [157,158]. More recently, Dzanaeva et al. [159] evaluated the impact of transcription factors Znf1, Sip4, Adr1, Tup1, and Hap4 on xylose catabolism in the xylose-fermenting strain of *S. cerevisiae*. The increase of ethanol production from xylose compared to that of parental strain confirms their involvement in regulating xylose growth and fermentation.

Prompted by the accomplishments with ethanol producer *S. cerevisiae*, the nonconventional yeast *Spathaspora passalidarum*, and *Pichia stipites* have also been explored for natural and engineered xylose metabolism to improve bioethanol production [160]. These yeasts have several novel alcohol dehydrogenase (ADH) encoding genes (PsADH1 to PsADH7 and SpADH1) essential for xylose assimilation and ethanol production. Interestingly, PsADH1 shows a broad spectrum for substrate-specificity and can also utilize xylitol as a substrate with moderate activity [161]. The oleaginous yeast, *Yarrowia lipolytica*, which only has native pentose-specific transporters (TRP6 and TRP22), is also being exploited to engineer xylose metabolism. Overexpression of these native transporters with xylitol dehydrogenase improves dxylose utilization and metabolism [162]. This species has extensively been used to engineer lipid accumulation to enhance the biosynthesis of biodiesel and other lipid-derived products [163,164]. The subcellular engineering of lipase-dependent pathways in *Y. lipolytica* resulted from 14-folds increased lipid titer [165]. Yook et al. [166] developed an efficient xylose-utilizing *Y. lipolytica* strain through CRISPR–Cas9-mediated rational and evolutionary engineering. This isomerase-based xylose xylose-utilizing strain, YSXID, produced 12.01 g/L lipids with a maximum yield of 0.16 g/g, the highest ever reported, from lignocellulosic hydrolysates. In the oleaginous yeast domain, *Rhodosporidium toruloides* can also utilize carbon sources (apart from sugars) available in lignocellulosic hydrolysates, such as organic acids lignin-derived phenolic compounds [167,168]. Some engineering has been implemented on this species to improve lipid conversion and productivity. Diaz et al. [169] implemented combining evolutionary and metabolic engineering to *R. toruloides* and reported high lipid yield (0.179 g/g) with non-detoxified lignocellulosic hydrolysates. Increased lipid production was also observed in *R. Toruloides* strains by overexpressing gene encodes Δ9 fatty acid desaturase (Δ9FAD), which synthesize palmitoleic and oleic acids [170]. Recently, Chopra et al. [171] reported increased lipid production via applying diverse cultivation practices to *R. toruloides* strains.

In addition, the robustness of yeast strains against toxic fermentation inhibitors (i.e., lignin-derived phenolics, organic acids, and sugar degradation products) present in lignocellulosic hydrolysates is crucial for the sustainability of biofuels. The robustness of model yeast, *S. cerevisiae*, has been extensively improved through several metabolic and evolutionary engineering approaches [172]. The tolerance of other ethanol-produced yeasts such as *S. passalidarum* and *S. stipites* to fermentation inhibitors has not been as extensively studied as that of *S. cerevisiae*. Although their robustness relies heavily on the type and level of inhibitors present in the hydrolysates [173,174], these species display substantial tolerance. The robustness of *Y. lipolytica* to lignocellulosic fermentation inhibitors has also been improved through metabolic engineering approaches [175,176]. The overpressing of the native genes XR, XDH, and XK also improved tolerance against formic acid, furfural, and coniferyl aldehyde [177]. The engineering efforts were performed to improve the overall biofuel production capacity of diverse yeast strains (Table 4).

**Table 4.** Summary of metabolic/genetic engineering approaches to advancing biofuel production from lignocellulosic hydrolyzates by yeasts strain

Species or Strain	Targeted traits	Genes	Outcomes	References
***S. cerevisiae***	Xylose metabolism	SIP4, ADR1 and HAP4	Increased ethanol yield (1.8 fold)	[159]
		SFA1	Increased ethanol yield (0.492 g/g total sugars) within 48 h	[154]
		ΔPMR1, ΔASC1	Increased ethanol titer (2–3 fold)	[152]
		PMR1	Enhanced ethanol concentration (3–4 fold)	[250]
		ΔPHO13, TAL1	Increased xylose utilization rate (3.4 fold)	[251]
	Xylose transport	HXK2	Increased xylose consumption rate (23.5%), ethanol production rate (78.6%), and the ethanol yield (42.6%)	[167]
		XK, XR, XDH	Increased xylose consumption rate (4.5 fold), and the ethanol yield (0.38 g/g total sugars)	[252]
		CYC8 or SSN6	Improved xylose uptake rate (1.5 fold)	[157]
		HXT2	Increased ethanol productivity (1.2 fold)	[253]
	Ethanol production Acetate utilization	AN25	Improved xylose uptake capability (43fold)	[156]
		PHO4	Increased ethanol yield (5.3%); reduced fermentation time (12.5%)	[254]
		Δssk1Δsmp1	6% increase in ethanol yield	[153]
		GndA	Increased ethanol yield on glucose (13%)	[255]
		EhADH1	Increased ethanol yield on glucose (4%)	[256]
	Inhibitortolerances	ARI1, PAD1 or TAL1and ADH6, FDH1 or ICT1	Increased inhibitor resistance	[21]
		HAA1	Improved tolerance against acetic acid	[257]
		RPB7	Increased ethanol titer (40%)	[172]
*Spathaspora passalidarum*		-	Improved xylose consumption rate (0.4 g/g), and ethanol productivity (19.4 g/l)	[174]
*Scheffersomyces stipitis*		-	Increased xylose consumption (25%) and ethanol yield (5%)	[173]
*Y. lipolytica*		XR, XDH, and XK	Improved inhibitor tolerance	[177]
		YALI0_E25201g, YALI0_F05984g, YALI0_B18854g, and YALI0_F16731g	Enhanced tolerance to ferulic acid	[176]
	Heterologous xylose catabolic pathway	Δpex10, DGA1, XylA, XK	Improved lipid titer (~10 fold)	[166]
		ylXDH, ylXR,ylXK, anXPKA, and anACK	Increased lipid titer (1.6 fold)	[258]
		ssXYL1, ssXYL2	29.3% of theoretical lipid yield	[259]
	Lipidaccumulationcapacity	TGL, CAR, ADC, OleTJE, ACC, GPD1, DGA1, ΔGUT2, ΔMFE1	Increased lipid titer (14 fold)	[165]
		-	Improved lipid yield (30%)	[260]
		SCD, ACC1, DGA1	Increased ethanol yield on glucose (2.93 fold)	[261]
*Rhodosporidium toruloides*		-	Increased lipid content (7.8%)	[262]
		ScOLE1, RtΔ9FAD	Increased lipid titer (5 fold)	[170]
		-	Increased lipid titer (~1.4 fold)	[263]
		DGAT1 and SCD1	Improved lipid yield (0.179 g/g)	[169]
		ACC1, DGA1	Increased lipid yield on glucose (~2 fold) and xylose (1.4 fold)	[167]

### 4.4. Fungi

Unsaturated fatty acids are desirable starting material for production of biodiesel. Ascomycota and Mucoromycota are major phyla comprising of oleaginous filamentous fungi capable of production of single-cell oils approximately 15–36% cell dry weight. Single-cell oils rich in monounsaturated fatty acids and saturated fatty acids can be utilized for production of third generation biodiesel. So, lots of work has been done to optimize the culture conditions for maximum production of single-cell oils from *Mortierella, Mucor*, and *Aspergillus Cunninghamella* [178]. Several metabolic engineering strategies for increased accumulation of lipid from oleaginous mucors have been adopted by several workers. 340 mg/l steariodonic acid was achieved by overexpression of fad3 gene (coding for fatty acid desaturase 3) in *Mucor circinelloides* [179] *Ashbya gossypii* mainly used for industrial production of riboflavin, is being engineered for increased production of lipid droplets also. Lipid production can be increased by providing lipid precursor and blocking the other competing pathways of lipid accumulation. A knock out in Ag*POX*1 gene resulted in blocking of β-oxidation pathway and increase the yield (approximately 70% of its total cell dry weight) of total lipids, additionally the culture medium was supplied with 1% glucose and 2% oleic acid to prevent the above pathway completely [180]. In the direction to increase the lipid accumulation some lignocellulosic hydrolysates, were also tested with 11 oleaginous fungal strains mainly including *Mortierella, Aspergillus,* and *Cunninghamella*. In the form of lignocellulosic hydrolysate substrate, mild sulphuric acid treated wheat straw provided sufficient xylose, on which *Mortierella isabelline* can grow and accumulate high amount of lipid (39.4%) [181]. The fungus mainly accumulates long-chain fatty acids, while the medium chain fatty acids are desirable starting material for biofuel production. To overcome this problem one of the fungi *Mucor circinelloides* has been genetically modified. The β- oxidation pathway was modified by integrating heterologous acyl-ACP thioesterase into Fatty acid synthetase complex with a sequential knockout of acyl-CoA thioesterase and/or acyl-Co-A oxidase genes. A total increase in accumulation of medium chain fatty acids was reported around 47.45% in compare to wild strain, which can accumulate only 2.25% [182,183].

Nanostructures are very much important for immobilization of various fungal strain in packed bed reactors, to improve the yield of hydrolyzing enzymes. More than 90% yield of biodiesel was reported in *Candida rugosa, Rhizomucor miehei*, etc., through the immobilization of these strains in nanostructures [184]. Besides Yeast, being a good eukaryotic system for metabolic engineering, other fungi such as *Trichoderma* spp. and *Aspergillus* spp. are main source of cellulase enzyme production at industrial scale. *T. reesei* has higher cellulase content but due to lack of β-glucosidase production co-culture with *A. phoenicis* gave a 2.5-fold increase β-glucosidase production [185]. In a different investigation, repertoire of proteins in the secretome of a catabolite repressor-deficient strain of *Penicillium funiculosum*, PfMig1 88, was successfully evaluated to enhance the saccharification of sugarcane bagasse [186].

## 5. Recent advances in microbial cell surface engineering for biofuel process

**Figure 4.**
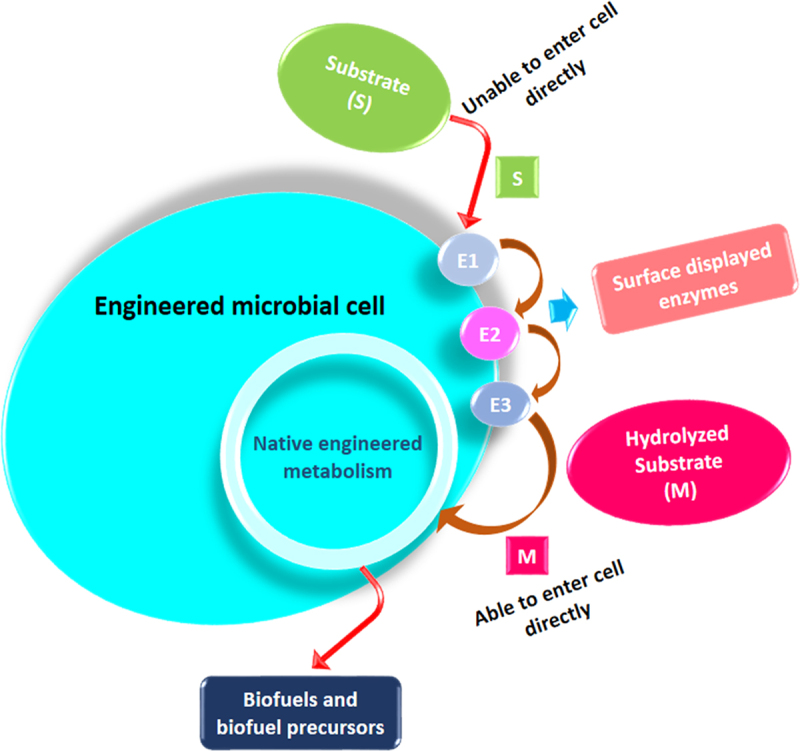
A model of metabolically engineered microbial cell with surface-displayed enzymes E1–E3.

Microbial cell surface engineering is an innovative technique to endow novel functions on host cells through displaying functional proteins or enzymes on the cell surface [187]. This approach has diverse strategies that involve communicating hydrolytic enzymes on the surface of microbial strains to degrade the LCS. Metabolically convert the degraded sugars directly into biofuels and biofuel precursors, thus elevating the status of microorganisms from immobilization stuff to a novel whole-cell biocatalyst (Figure 4). Yeast, mainly *S. cerevisiae*, is the major mainstay of this approach. Its surface display was first explored to develop ‘arming yeast’ that can be a good platform for self-immobilized biocatalysts [188]. Soon after, similar display systems were also employed to transform other yeast species, such as *P. pastoris* and *Y. Lipolytica* [189,190]. In the last couple of years, various studies demonstrated the significance of microbial cell surface display employed to design and optimize biofuel production from the LCS [191–193]. It has been shown that the use of yeast surface display to the lignocellulolytic enzymes, such as endoglucanase, cellobiohydrolase, β-glucosidase, etc., exhibited better hydrolytic activities than of their free enzyme counterpart [194,195]. Substantial development has also been reported in the degree of synergy if these surface-displayed enzymes are used in combination for substrate hydrolysis [196].

Interestingly, certain microbial clostridia can produce cellulosome, a complex of scaffolding proteins that requires multiple enzymes for efficient hydrolysis. Goyal et al. [197] developed a functional display of trifunctional minicellulosomes on the *S. cerevisiae*. They obtained 3-fold higher ethanol production than a similar yeast consortium secreting only the three cellulases on phosphoric acid swollen cellulose. Similarly, enhancement of hydrolytic activity with improved ethanol titer has been reported while a bi-functional minicellulosome was accomplished on *S. Cerevisiae* [198]. A cellulosome with a multiple-component assembly system was also developed through disulfide bonds. This cellulosome complex enabled yeast to ferment cellulose into ethanol proficiently [192] directly. Recently, Dong et al. [199] engineered *P. pastoris* to display minicellulosomes. The incubation of the protein-displaying yeast with three recombinant cellulases, including endoglucanase, an exoglucanase, and a β-glucosidase fused with a carbohydrate-binding module led to a cellulosome displaying *P. pastoris* able to direct conversion of carboxymethyl cellulose to bioethanol, observing an impressive ethanol titer of 5.1 g/L. The surface display engineering approaches employed on various microbial consortiums have been attempted to hydrolyze other lignocellulosic components, including xylan and lignin [200–202]. Table 5 summarizes the applications of microbial cell surface display during biofuel production.

**Table 5.** Applications of microbial cell surface display during biofuel production

Name of microorganism	Applied strategy	Substrate	Product	Advancement	Reference
*S. cerevisiae*	Expression of co-displaying endoglucanase and β-glucosidase	Corn cob	Ethanol	High ethanol titers (>50 g/L)	[24]
	Expression of co-displaying endoglucanase, β-glucosidase, cellobiohydrolase I and II, xylanase, β-xylosidase and acetylxylan esterase	Lignocellulosic substrate	Ethanol	-	[195]
	Display of bifunctional mini cellulosomes by galactose induction and a cellodextrin pathway	Cellulose	Ethanol	Higher ethanol yield of 0.43 g/g of total sugars	[264]
	Expression of phytase utilizing the C-terminal half of the yeast αagglutinin protein	Corn substrate	Ethanol	1.04-fold higher ethanol production that of conventional strain	[265]
	Expression of heterologous endoglucanase and cellobiohydrolase co-displaying β-glucosidase	Lignocellulosic substrate	Ethanol	High ethanol yield	[194]
	Co-displaying endoglucanase II and β-glucosidase	Cellulose	Ethanol	106-fold higher hydrolysis activity that of conventional strain	[[266]
	Co-expressing of cellulase and expansin-like protein	Cellulose	Ethanol	1.4-fold higher that of conventional strain	[267]
*Pichia pastoris*	Displaying minicellulosomes combining with endoglucanase, exoglucanase, a β-glucosidase and carbohydrate-binding module	Cellulose	Ethanol	Ethanol titer of 5.1 g/l	[199]
*E. coli*	Expression of displaying α-amylase	Starch	Ethanol and hydrogen(H_2_)	H_2_ (1689 cm^3^/dm^3^) and ethanol (2.8 g/dm3) production	[193]
	Expression of co-displaying lipase, carboxylic acid reductase and aldehyde reductase	Lignocellulosic substrate	Fatty alcohol	High conversion rate (73%)	[268]
	Co-expressing of cytosolic and outer-membrane-targeted (osmoregulatory membrane protein, OmpC,) fused tilapia metallo thioneins (TMT)	Lignocellulosic substrate	n-butanol	Improved n-butanol productivity (up to 320 mg/l)	[196]
	Expression of co-displaying type V secretion system (TVSS) and β-glucosidase	Cellobiose	Ethanol	Ethanol yield of 81% of the theoretical maximum	[269]
	Expression of co-displaying β-glucosidase and anchor protein Blc	Cellobiose	Isopropanol	Improved yield	[270]
***Synechocystis sp.***	Expression of co-displaying four Calvin-Benson-Bassham cycle enzymes and pyruvate decarboxylase (PDC) and alcohol dehydrogenase (ADH)	Lignocellulosic substrate	Ethanol	33–69% more ethanol	[23]

## 6. Recently enabled technological advancement for biofuel processes

**Figure 5.**
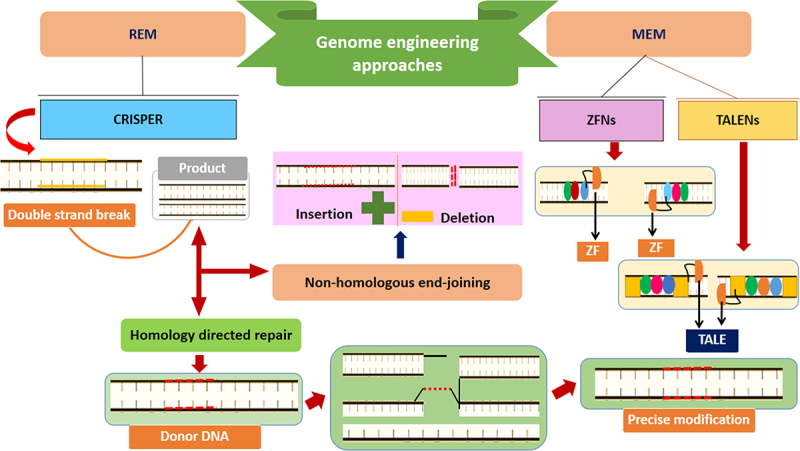
Overview of genome engineering strategies for microbial biofuel production, MEM = modified en-donuclease-mediated engineering, REM = RNA-guided endonuclease-mediate, TALENs = transcription activator-like effector nucleases, ZFNs = zinc-finger nucleases, ZF = zinc-finger, TALE = transcription activator-like effector.

Quantification and regulation of the metabolic flux and metabolic pathway are crucial for optimizing the microbial biofuel production processes [203]. Biosensors, genetically encoded compounds that convert input signals to a measurable output (i.e., gene expression, fluorescence, etc.), have been included recently among persuasive tools in the metabolic engineering field [204,205]. They have more comprehensive applications in biofuel production processes, from increasing substrate utilization and precursor accessibility to optimizing the product titers and activating pathways to *in vivo* monitoring of the target compounds [206]. Several studies regarding metabolic engineering exploited biosensors to optimize the biofuel products, such as 2-ketoisovalerate [207], isoprene [208], fatty acids [209], butanol and alkenes [210], and fatty alcohol [211]. More advanced gains in optimizing these processes can be achieved through efficient genetic engineering/editing tools, including CRISPR, to improve cell metabolism [212]. There are two key genome editing tools/methods: modified endonuclease-mediated (MEM) engineering and RNA-guided endonuclease-mediate (REM) engineering (Figure 5). Researchers in this field have experienced a dramatic revolution through such methods, and numerous challenges in the metabolic issues of biofuel production have been positively revolutionized during the last few years [25,213,214]. Recent efforts have also been made to express new predictable and controllable multiplex genes and optimize such tools with high-throughput editing and efficiency of the genes [215,216].

## 7. Recent patents on metabolic engineering of microorganism for biomass conversion to biofuels

Protection of intellectual property (IP) related to the strategies and methods to improve biofuel production by modifying microorganisms is under intense development. Several recombinant *Z. mobilis* or their allied species express xylanases that enable pentose sugars’ catabolism with improved ethanol production [217,218] and synthesize butanol and/or isomers of butanol [29] have been patented. Recombinant yeast that can ferment pentose sugars [219] restrained xylitol [220] and that synthesize ethanol and fatty alcohols [221,222] have also been patented. The University of California, United States, recently filed a patent describing biofuel production by recombinant microorganisms [223]. This patent describes metabolically modified microorganisms helpful in producing biofuels, more specifically 1-butanol, 1-propanol, 2-methyl-1-butanol, 3-methyl-1-butanol, and 2-phenylethanol from a suitable substrate. More recently, the trustees of three universities of the US (Princeton University, Massachusetts Institute of Technology, Whitehead Institute for Biomedical Research), and the Kyoto University of Japan filed a patent application [224] that describes recombinant yeast that overproduces the heavy alcohol, mainly branched-chain alcohols. This yeast strain contains at least one deletion, disruption, or mutation from the individual GLN, VPS, GNP, AVT, GCN, or YDR391C gene families and combinations thereof. Some of the recent patents on the modification of microorganisms for advancing biofuel production (Table 6). In addition, Algenol Biofuels and Joule Unlimited, US biofuels companies, have made significant contributions in photoautotrophic ethanol production using *Synechococcus* 7942 and *Synechocystis 6803* systems. Algenol has filled various patent starting from 1998, publication number US6306639B1, followed by US8163516B2 (filed 9 February 2009), which was in itself first patent showing increased ethanol yields via overexpression of an alcohol dehydrogenase [225] Further, in recent years filed a patent dealing with Cynobacteria (US20140113342A1) for production of 1, 2-propanediol with yields of ~0.011 g L-1[226]. The Joule Unlimited patent space target wider metabolite profile than Algenol Biofuels, comprising of supplier photosynthetic framework for production of various industrially important metabolites. In their one of the patents using JCC1581_B isolate (patent application US20120164705A1, (filed 13 November 2011), yield of 5.62 g/l after 13.7 days was achieved, which was the higher ever reported yield for the *Synechococcus* 7002 framework [227].

**Table 6.** Recent patents on modification of microorganisms for advancing biofuel production

Patent #	Patent Owners	Inventors	Patent Title	Date publication/filed
WO2021062082	The Trustees of Princeton University, NJ (United States), Massachusetts Institute of Technology, MA (United States), Whitehead Institute for Biomedical Research, MA (United States) and Kyoto University, Japan	Avalos et al. [271]	System and method for increased alcohol tolerance and production in yeast	2021–04-01
US20190153483A1	Alliance for Sustainable Energy LLC	Zhang et al. [29]	Engineered *Zymomonas* for the production of 2,3-butanediol	2021–08-17
US20210017526A1	BASF Corporation, New Jersey	Xu et al. [219]	Xylose metabolizing yeast	2021–01-21
JP2020115827A	Nippon Oil & Energy Corp, Japan	Konishi et al [220].	Yeast with inhibited accumulation of xylitol	2020–08-06
US10557152B2	University of California, Oakland, CA (United States)	D<apos;>espaux and Keasling [222]	Yeast host cells and methods for producing fatty alcohols	2020–02-11
JP2020025493A	Toyota Motor Corporation	Onishi and Tada [223]	Recombinant yeast, and method for producing ethanol using same	2020–02-20
JP6616311B2	JXTG Energy Co., Ltd. Japan	Konishi et al. [221]	Yeast producing ethanol from xylose	2019–12-04
EP3160987B1	EI Du Pont de Nemours and Company, LLC	Eliot et al. [218]	Enhancing d-xylose and l-arabinose utilization in *Zymomonas* cells	2018–10-17
US20170183670A1	Massachusetts Institute of Technology, Cambridge, MA (United States)	Stephanopoulos et al. [272]	Strain and bioprocess engineering for high lipid production	2017–04-27
US9,695,426	University of California, Oakland, CA (United States)	Liao et al. [224]	Biofuel production by recombinant microorganisms	2017–07-04
CN105886524A	Jiangnan University, Wuxi (China)	Song and Zhang [273]	Method for raising *Yarrowia lipolytica* lipid content by molecular modification	2016–08-24
ES2554805T3	EI Dupont Doe Nemours and Company, LLC	Viitanen et al. [217]	*Zymomonas* xylitol synthesis mutant that uses xylose for ethanol production	2015–12-23
US9127297B2	Algenol Biotech, LLC	Dühring et al. [274]	Metabolically enhanced *Cyanobacterial* cell for the production of ethanol	2015–09-08

## 8. Safety and hazard concerns

Recombinant microorganisms have opened new alternatives for cleaner and more efficient production of various metabolites including biofuels. Still, high cost, low efficiency of raw material conversion and unintentionally release of genetically modified microorganisms in the environment have limited the use of bioprocess using living cells and cell-free metabolic engineering becoming popular to harnesses the metabolic activities of cell lysates *in vitro* [228]. Similarly, compound toxicity in bacteria becoming a major concern due to accumulation of toxic compounds during expression of heterologous biosynthetic pathway. The production of desired compound can be limited, on the other hand, the toxic intermediates can be useful as important antimicrobial compounds [229]. The genetically modified microorganisms can have effect on indigenous community of natural microorganisms by creating more vigorous strains, overpowering the existing strains by recombination and potent gene exchange, loss of genetic pool, narrowing genetic diversity, permanent loss of primary metabolic pathway that can lead to loss of accumulation/production of important secondary metabolites [230]. Though the gene exchange by natural recombination process is occurring at its own pace, still the unknown consequences are expected by foreign gene expression and results in altered metabolic pathway, growth rate, and response to external environmental factors [231,232].

Environmental risk assessment of various genetically modified organisms (GMO) has been done time to time [233–235] but still there is a lack of specific rules and regulatory bodies regarding production of value added by products through genetically modified microorganisms (GMMO). There is a need to emphasize and differentiate the specific assessment criteria for both GMMOs and GMOs. The environmental risk assessment area is vast and open to explore the overall performance of GMMOs.

## 9. Conclusions and future prospects

Through the application of metabolic and bioprocess engineering approaches, numerous challenges associated with the production of lignocellulosic biofuels have been successfully revolutionized during the past few years. In brief, improvements in the biosynthesis of polysaccharides, lignin, and lipids in the lignocellulosic biomass have led to limited success, and further progress is required for sustainable biofuels. Metabolic pathways of microbial strain from diverse domains, such as yeasts, bacteria, and cyanobacteria have been successfully engineered to produce bioethanol from lignocellulose with a high yield near its maximum theoretical yield (0.51 g/g glucose). Yeasts, mainly *S. cerevisiae*, are already being engineered and commercialized to ferment simple sugars to isobutanol and other alcohols. Some non-conventiona,l such as *S. passalidarum, P. stipites*, etc., and a few oleaginous yeasts, i.e., *Y. lipolytica, R. toruloides* are being engineered to produce both bioethanol and biodiesel. Yeast strains engineered to surface display hydrolytic enzymes appear to be much more promising and have a broader prospect for commercial-scale bioethanol production directly from LCB in upcoming years. The bacterium *Z. mobilis, E. coli*, and other thermophiles are being engineered to ferment the xylose and could be used in commercial processes for biofuels production from lignocellulosic hydrolysates. Cyanobacteria and other photosynthetic bacteria also seem to generate commercializable biofuels in the future potentially. Although metabolic engineering is a well-established strategy for transforming microorganisms into efficient cell factories, there is a high possibility that novel robust industrial phenotypes could emerge from the coupling of more database mining, omics technologies, and advanced genome engineering strategies. Futuristic, metabolic engineering of microorganisms to discover new enzymes catalyzing unknown reactions, engineered enzymes with enhanced performances, and new synthetic pathways designed for higher metabolic flux towards target biofuels and other value-added products may enable the production of more sustainable biofuels.
